# Reducing thrombotic risks in video gamers: investigating the benefits of walking and compression sleeves on blood hemodynamics

**DOI:** 10.1152/ajpheart.00669.2023

**Published:** 2023-12-22

**Authors:** Joanne DiFrancisco-Donoghue, Kelly Borges, Timothy Li, Olivia Ballone, Hallie Zwibel, Peter C. Douris

**Affiliations:** ^1^Department of Osteopathic Medicine, New York Institute of Technology College of Osteopathic Medicine, Old Westbury, New York, United States; ^2^Medical School, New York Institute of Technology College of Osteopathic Medicine, Old Westbury, New York, United States; ^3^Department of Family Medicine, New York Institute of Technology College of Osteopathic Medicine, Old Westbury, New York, United States; ^4^Department of Physical Therapy, New York Institute of Technology, Old Westbury, New York, United States; ^5^Center for Esports Medicine, New York Institute of Technology, Old Westbury, New York, United States

**Keywords:** blood flow, deep vein thrombosis, DVT, esports, prolonged sitting

## Abstract

With the growing popularity of video gaming, deep vein thromboses are increasingly being reported in gamers. This study aimed to compare the effects of lower leg graduated compression sleeves and a 6-min walking break during prolonged gaming on blood flow and hemodynamics in competitive sport players to help mitigate this risk. Ten healthy gamers (19.6 ± 1.2 yr old; 9 men) consented to participate in this mixed-model crossover design study that consisted of three visits. In *visit 1*, participants engaged in continuous 2-h video game play wearing no compression (continuous). *Visits 2* and *3* involved 2-h play wearing compression sleeves (compression) and 2-h game play interrupted at 1 h by a 6-min walk (walk). Doppler ultrasound measurements of the left popliteal artery were taken at 30, 60, 90, and 120 min, to record vessel diameter, blood flow velocity, and blood flow volume. Participants completed a survey to assess their perception of each approach. There was a significant interaction between conditions for blood flow and blood velocity (*P* = 0.01, *P* < 0.001). Post hoc analysis demonstrated a greater decrease in blood flow and blood velocity in the continuous group compared with the walk group at the 90-min mark (*P* = 0.04, *P* = 0.01). No differences were found between the compression and walk groups or between the continuous and compression groups (*P* = 0.42, *P* = 0.69). No interactions were observed in diameter, mean arterial pressure, or heart rate. This study suggests that incorporating a 6-min walk every 60 min during prolonged gaming is advisable to counteract the negative effects on blood flow hemodynamics.

**NEW & NOTEWORTHY** A 6-min light-intensity walking break during gaming can effectively combat the adverse effects of prolonged sitting, surpassing compression garments. Prolonged sitting reduces blood flow velocity, potentially leading to deep vein thrombosis (DVT). Compression sleeves help, with superior results after a 6-min walk at 60 min. Although compression stockings offer moderate improvements, a 6-min active break proves more effective. These findings offer promising interventions for gamers’ health, initiating guidelines to mitigate DVT risk during gaming.

Listen to this article’s corresponding podcast at https://ajpheart.podbean.com/e/reducing-thrombotic-risks-in-video-gamers/.

## Graphical abstract

**Figure:**
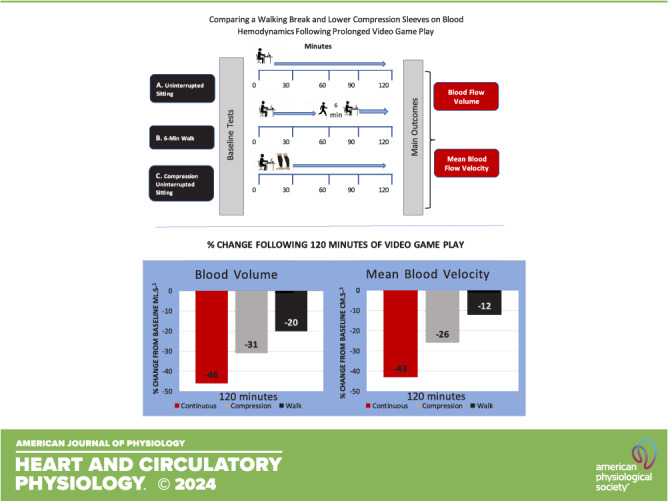

## INTRODUCTION

Deep vein thrombosis (DVT) and pulmonary embolism (PE) are associated with prolonged sitting and immobility. A DVT typically forms in the leg or pelvic veins and can become life threatening as it can travel to the lungs, potentially leading to a PE and death (1–3). “E-thrombosis” is a term proposed by Dr. Beasley 2 decades ago, hypothesizing that the increase in computer use for work and decreased physical activity would lead to an increase in DVTs and PEs brought on by immobility and prolonged sit time (4). The recognition of immobility and its potential health hazards, including PE, was already acknowledged as far back as World War II, when people were forced to sleep in a sitting position on deck chairs for prolonged periods during air raids, which tragically resulted in fatal outcomes for certain individuals (5). Fast forward to the present, where esports and video game use have exploded at an astronomical rate, and Dr. Beasley’s proposed theory has been supported by many case studies and clinical studies documenting computer use as a major contributor to DVT and PE. There have been >22 documented cases of DVTs and PE brought on by excessive gaming in the past few years in young adults (6). Although DVTs are not the norm in younger adults or children, gamers have nearly double the risk of developing a thrombolytic event compared with nongamers (7).

### Pathophysiology

A triad of physiological changes during prolonged sitting have been proposed that increase the risk of DVT. The first is changes in stasis or low blood flow, the second is changes in the blood itself, and last is the integrity of the endothelial lining of the blood vessels (8). However, endothelial dysfunction and impaired venous return are believed to be a primary contributor to the development of atherosclerotic lesions long term and the primary cause for DVTs in the near term (9, 10). In the rapidly evolving field of competitive esports, what are particularly noteworthy are the implications of heart rate variability (HRV) and sympathetic nervous system activity, especially regarding their influence on hemodynamics, including vasoconstriction. Unlike traditional athletes, esports players engage in prolonged periods of intense mental exertion and minimal physical activity, a combination that yields unique physiological responses. Sympathetic nervous system activity, typically heightened during competitive gaming, can induce vasoconstriction, thereby impacting blood flow and pressure. (11–13) This heightened state of alertness, beneficial in short bursts, can, over extended periods, lead to increased cardiovascular strain. This is especially pertinent in high-stake scenarios common in esports, where prolonged sympathetic activation may exacerbate stress responses and potentially lead to sustained increases in heart rate (HR) and blood pressure.

### Prolonged Gaming Implications

The detrimental effects of chronic extended periods of sitting have been a health concern for the medical profession for many years. However, because of the recent surge in popularity of esports and video game play, the health risks associated with prolonged sitting affect a wider and younger demographic beyond just office workers. Several studies have shown that short, frequent breaks improve blood flow (3, 14–17). Individuals can enhance blood circulation, and possibly mitigate the potential risks associated with extended periods of sedentary behavior, by engaging in regular breaks (3, 15–17).

### Compression Stockings

Compression garments are used to exert external pressure on the lower extremities to reduce vascular wall tension and prevent gravity from pooling blood in the lower extremities, which improves venous return and lymphatic output. Air travel and prolonged immobility have long been associated with an increased risk of DVT, earning the term “economy class syndrome” (3, 18, 19). Graduated compression stockings below the knee are recommended for patients at high risk to prevent edema and DVT (20). Research supports wearing compression garments during air travel, with studies showing a 19-fold reduction in DVT risk by reducing vessel diameter and increasing blood velocity (3). Given the nature of esports, sitting for prolonged periods, graduated compression wear may offer significant health benefits.

The aim of the present study was to investigate two techniques for enhancing blood flow and hemodynamics in individuals engaged in extended gaming sessions. The main objective was to contrast the impact of a brief walk with the application of graduated compression sleeves on blood flow dynamics in competitive gamers engaged in extended sitting. This study hypothesizes that in individuals engaged in extended gaming sessions specific interventions aimed at enhancing blood flow and hemodynamics, namely, brief walking breaks and the use of graduated compression sleeves, will demonstrate distinct effects on blood flow dynamics. We anticipate that both interventions will positively impact blood hemodynamics and reduce the risk of DVT and PE associated with prolonged immobility. However, the magnitude and nature of these effects are expected to differ between the two interventions. Additionally, this study seeks to assess the subjective perceptions of competitive gamers regarding the efficacy and practicality of these interventions during extended gaming sessions. We hypothesize that these interventions will not only improve physiological outcomes but also be positively received by the gaming community, thus offering practical and effective strategies to mitigate the health risks associated with prolonged sedentary behavior in esports athletes.

## METHODS

### Study Design

This study was a mixed-methods design with a repeated-measures randomized crossover design within-group measure over time (0, 30, 60, 90, and 120 min), a between-group measure in a continuous play group (continuous), sitting with compression (compression), and sitting with a walk break (walk), and an exit survey after each intervention.

### Participants

**Figure 1.**
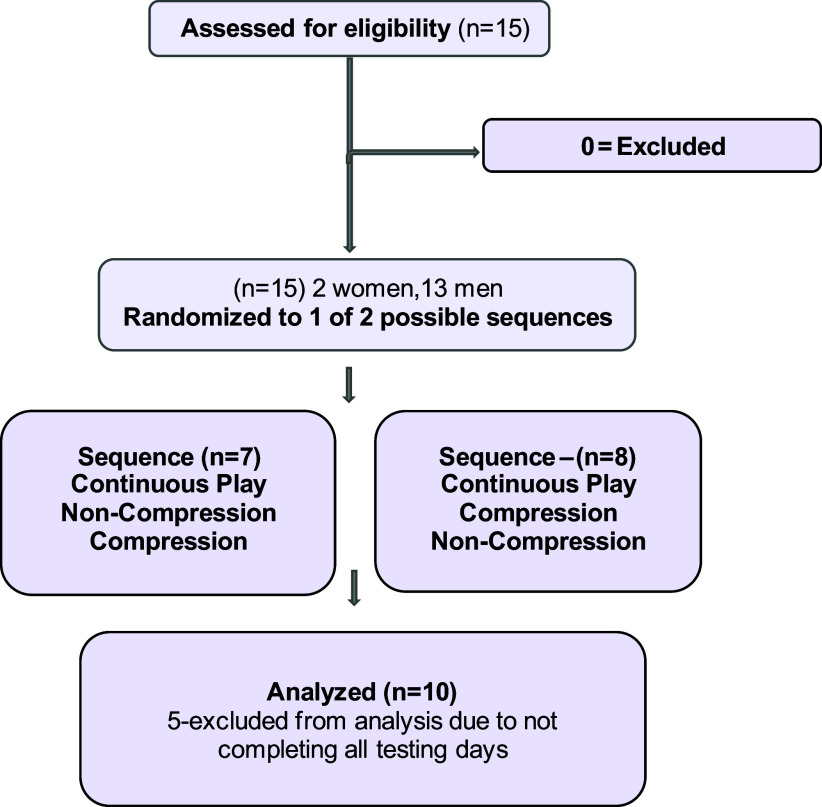
Study flow.

This study was approved by the New York Institute of Technology (NYIT) Institutional Review Board (BHS-1664) and was registered on clinicaltrials.gov (NCT05212363). All subjects gave written informed consent before participation, and the study was carried out in accordance with the Code of Ethics of the World Medical Association (Declaration of Helsinki 1964 and Declaration of Tokyo 1975, as revised in 1983). The determination of the sample size for this study was influenced by findings from a previous study conducted by Thosar et al. (17). Based on these results, and aiming for an 80% statistical power and a significance level set at *P* = 0.05, we calculated that a total of 10 subjects would be sufficient for our randomized, crossover design study (17). Twelve healthy men and women first-person shooter gamers between the ages of 18 and 22 yr old participated in this study; two subjects withdrew because of personal reasons (Fig. 1). A total of 10 subjects completed the protocol (Fig. 1). Inclusion criteria consisted of *1*) ranked esport player with >500 h in their game; *2*) nonsmoker; *3*) no prior history of heart disease, pulmonary disease, or metabolic disease including diabetes; and *4*) not using any over-the-counter or prescription drugs that might affect blood viscosity or metabolic results. This was determined by a detailed health history questionnaire. Exclusion criteria included *1*) peripheral neuropathy or any other condition that impacts skin sensation, *2*) a history of peripheral arterial bypass grafting, *3*) peripheral artery disease, *4*) skin infection, *5*) dermatitis with oozing or fragile skin, *6*) current leg swelling, or *7*) pulmonary edema.

### Procedures

The subjects reported to the NYIT esport gaming laboratory in Old Westbury, New York, for 3 testing days lasting 2.5 h. Subjects arrived at least 1 h postprandially. The room was kept temperature controlled within 2–3 degrees of 21°C each testing day.

All subjects rested quietly sitting in the gaming chair for 10 min. After the rest period, popliteal artery blood flow velocity, volume, and diameter were assessed at rest with a Philips IU22 Ultrasound System SN: B0MJ18 and Philips L9-3 Transducer SN: 03F40R (Koninklijke Philips). These measurements were performed again at 30, 60, 90, and 120 min (2 h). All measurements were conducted by a registered vascular technologist who was registered by the American Registry for Diagnostic Medical Sonography. To ensure accurate and consistent probe placement, subjects were seated with their knee slightly bent. The ultrasound imaging was employed to precisely identify and mark the placement of the popliteal artery. Measurements were recorded on the left side of the participant’s leg, in alignment with the approach used by Stebbings et al. (18). This was also done to avoid any potential interference with the right arm movements involved in using a mouse controller during gaming, ensuring an unhindered gaming experience.

#### Condition 1.

After the 10-min rest/measurement phase, all subjects remained seated and played continuously for 2 h with no break.

#### Condition 2.

The subjects sat for a 10-min rest phase for premeasurements and then gamed for 1 h. At the 60-min mark, the investigators timed the subjects as they were instructed to walk at a comfortable pace for 6 min. This was conducted next to the esport laboratory on a flat surface 50 ft in length. After the walk, the participants were shown the 6–20 BORG Rating of Perceived Exertion Scale (RPE). This RPE is a validated scale to measure how hard a person perceives they are working during physical activity and has been validated to correlate heart rate and perceived effort during active video game play (19). The subjects then played for another 60 min. We strategically chose a 6-min walk break at the 1-h mark during a 2-h gaming session, drawing upon the findings of two key pieces of prior research. This decision was primarily influenced by the work of Sousa et al. (20), who demonstrated the cognitive benefits of such an intervention. Specifically, Sousa et al. found that a 6-min walk, when integrated at the 1-h point in a gaming session, significantly improved executive function among collegiate gamers. This finding was crucial as it highlighted the potential of short walking breaks in enhancing cognitive performance, a key aspect for gamers.

Additionally, our choice was informed by the research of Thosar et al. (17), who investigated the impact of physical activity breaks on endothelial function during prolonged sitting. The study of Thosar et al. revealed that endothelial function, crucial for cardiovascular health, was maintained with 5-min walking breaks at intervals of 30, 90, and 150 min. This finding underscored the importance of incorporating physical activity into prolonged periods of sedentary behavior, like gaming, to preserve vascular health.

However, the practicalities of esports and gaming sessions necessitated a deviation from the 30-min interval proposed by Thosar et al. Most esports games last longer than 30 min, making a walking break every 30 min impractical and potentially disruptive to the gaming experience. Therefore, aligning with the model of Sousa et al., we opted for a single break at the 60-min mark. This approach aimed to balance the cognitive and physiological benefits of physical activity, as evidenced in previous studies, with the practical constraints and flow of esports gaming sessions. The 6-min duration was chosen to provide a sufficient but concise period for physical activity, maximizing the benefits without significantly interrupting the gaming experience.

#### Condition 3.

The subjects were fitted with lower compression sleeves. They sat for a 10-min rest phase for sitting premeasurements. Subjects continued to play for 120 min with no active break while wearing the compression garments. Upon completion of testing on *day 3*, subjects were given a short survey regarding their perceptions of wearing compression garments and the walking condition.

**Figure 2.**
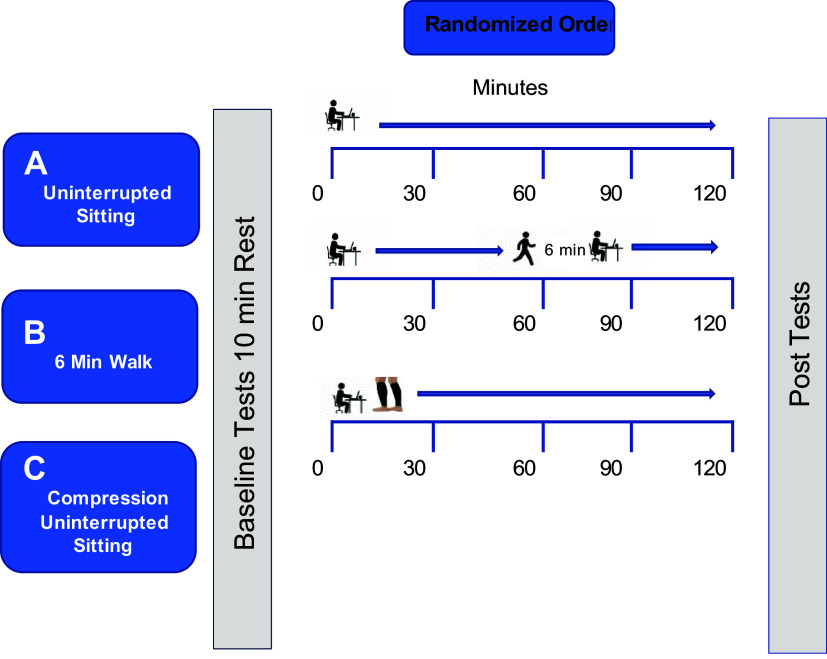
Study procedures.

Testing was randomized by subject in order of sequence regarding use of compression garment versus no compression (control continuous group) and a 6-min walk using an online sequence generator (21). (Fig. 2)

### Measuring for Proper Lower Body Compression

**Figure 3.**
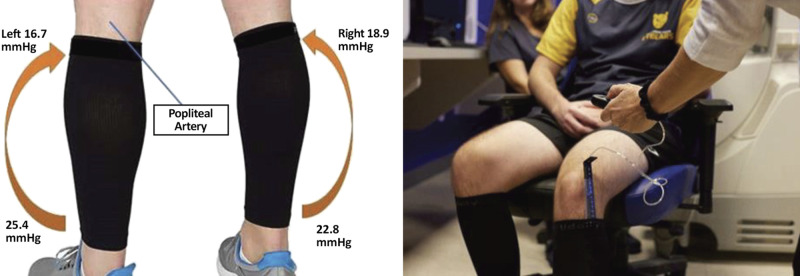
*Left*: graduated calf compression sleeves showcasing the average pressure used in this study. *Right*: Juzo compression sensor used for measuring compression pressure both distally and proximally.

This study used graduated compression garments that fit below the knee, fitted according to manufacturer instructions. Under the compression condition, a Juzo pressure monitor (Compression Innovations, Cuyahoga Galls, OH) was inserted under the compression garment to measure distal and proximal millimeters of mercury of pressure and pounds per square inch were recorded (Fig. 3).

### Statistical Analysis

All statistical analyses were performed with IBM SPSS V.28. A 3 × 4 multifactor repeated-measures analysis of variance was used to determine a main effect or interaction in each dependent variable. A Holm–Bonferroni post hoc analysis was conducted when significance was found. The assumption of sphericity was tested with Mauchly’s test. If sphericity was violated, a Greenhouse–Geisser correction factor was applied. All outcome measures were analyzed using the Delta of each dependent variable. Descriptive participant characteristics were done at baseline (Table 1). Statistical significance for this study was set at *P* ≤ 0.05. Descriptive statistics were also used to compare subject perceptions of each condition after the last testing day.

**Table 1.** Demographics

*n*	10
Age, yr	19.6 (1.2)
Weight, kg	76.1 (7.9)
Height, cm	176.0 (7.6)
Body mass index, kg/m^2^	24.7 (3.6)
Men, %	90
Right-handed, %	100
Ethnicity, %	
African American	10
Asian	30
Hispanic	20
Caucasian	40
Primary game (%)	
Halo	10
Valorant	20
Overwatch	20
Rocket league	20
Call of duty	30
Casual hours played weekly, %	
1–2 h	10
3–4 h	20
5–6 h	10
>6 h	60
Competitive hours played weekly, %	
1–2 h	40
3–4 h	30
5–6 h	30
Average play time before taking a break, min	93 (36)
Sleep average, h	6.6 (1.6)
Physical activity level (IPAQ-SF), %	
Low	10
Medium	50
High	40

Values are means (SD) or percentages. BMI, body mass index; IPAQ-SF, International Physical Activity Questionnaire-Short Form.

### Qualitative Analysis

After completing all three intervention arms, participants were asked to reflect on their perception of each intervention and how they felt it affected their gaming performance. The qualitative data from the exit survey were initially analyzed by using open coding to identify primary themes (22).

## RESULTS

Participants had a mean age of 19.6 ± 1.2 yr, a mean body weight of 76.1 ± 7.9 kg, height of 176.0 ± 7.6 cm, and body mass index (BMI) of 24.7 ± 3.6 kg/m^2^. Five participants were classified as healthy weight, four were classified as overweight, and one was classified as obese [according to the World Health Organization (WHO) BMI] (23). Participants self-reported 20 ± 16.9 h of sitting during a 7-day week; self-reported physical activity (PA) was 2,726.3 ± 1,210.5 min/wk. Based on the validated International Physical Activity Questionnaire (IPAQ) scoring (24), four participants were classified as having high PA levels, five moderate PA levels, and one low PA level (Table 1). The average intensity of the 6-min walk break was a reported RPE of 9.5 (very light intensity on the BORG 0–20 scale). The compression pressure measured at an average of 25.4 mmHg for left distal compression, 22.8 mmHg for right distal compression, 16.7 mmHg for left proximal compression, and 18.9 mmHg for right proximal compression.

### Superficial Popliteal Artery Hemodynamic Measures

All time point values for HR, mean arterial pressure (MAP), mean blood flow, mean blood velocity, and vessel diameter are presented in Table 2. A significant interaction was observed between condition and blood flow volume [*F*(6,48) = 3.49, *P* = 0.01], with post hoc analysis revealing a greater decrease in blood flow in the continuous group compared with the walk group at 90 min (*P* = 0.04) but no difference between compression and walk or between continuous and compression (*P* = 0.42; *P* = 0.69). A significant interaction was also observed in blood velocity between conditions [*F*(6,42) = 4.72, *P* < 0.001]. Post hoc analysis revealed a larger increase in blood velocity at 90 min in the walk group compared with the continuous group (*P* = 0.01), with no change between compression and continuous (*P* = 0.25) or between compression and walk (*P* = 0.47). There was no interaction observed for MAP (*P* = 0.61) or HR (*P* = 0.67). See Table 2 for all outcomes with confidence intervals.

**Table 2.** Change of study parameters over time

	Time									*P* Value (Within Group)
	0	30 min	Δ	60 min	Δ	90 min	Δ	120 min	Δ	
HR, beats/min										
Continuous	78.4 ± 11.7	80.3 ± 13.6	1.9 ± 9.5 [−4.0, 7.8]	80.1 ± 10.4	1.7 ± 10.1 [−4.7, 8.0]	80.6 ± 10.4	2.2 ± 10.9 [−4.6, 9.0]	74.2 ± 11.3	−4.2 ± 10.3 [−10.6, 2.2]	0.14
Compression	76.5 ± 4.2	77.1 ± 8.4	0.6 ± 7.3 [−4.0, 5.2]	77.0 ± 8.5	0.5 ± 7.0 [−3.8, 4.8]	75.5 ± 8.1	−1.0 ± 7.8 [−5.8, 3.9]	80.0 ± 7.6	3.5 ± 7.8 [−1.4, 8.3]	0.06
Walk	71.3 ± 10.3	70.1 ± 8.6	−1.2 ± 10.8 [−7.9, 5.6]	73.5 ± 10.8	2.2 ± 9.8 [−3.9, 8.3]	71.1 ± 9	−0.2 ± 10.2 [−6.4, 6.1]	73.4 ± 14.9	2.1 ± 12.4 [−5.6, 9.8]	0.57
MAP, mmHg										
Continuous	82.9 ± 4.6	73.4 ± 27	−9.5 ± 26.6 [−26.0, 7.0]	82.9 ± 9.2	−0.1 ± 10.2 [−6.4, 6.3]	88.5 ± 10	5.6 ± 7.2 [1.1, 10.1]	84.8 ± 8.5	1.9 ± 8.4 [−3.3, 7.1]	0.27
Compression	81.3 ± 6.1	83.7 ± 8.5	2.4 ± 8.7 [−3.3, 8.1]	84.9 ± 8.9	3.6 ± 10.0 [−2.6, 9.9]	81.7 ± 8.1	0.4 ± 8.2 [−4.7, 5.5]	86.3 ± 10.2	5.1 ± 12.3 [−4.0, 14.2]	0.47
Walk	82.5 ± 5	82.0 ± 6.9	−0.4 ± 8.2 [−5.6, 4.7]	81.5 ± 10.1	−1.0 ± 10.0 [−7.2, 5.2]	87.0 ± 15.3	4.5 ± 13.0 [−3.6, 12.6]	82.2 ± 8.5	−0.3 ± 7.1 [−4.7, 4.1]	0.99
Diameter, cm										
Continuous	0.61 ± 0.1	0.61 ± 0.04	−0.003 ± [−0.06,0.05]	0.6 ± 0.03	0.004 ± 0.01 [−0.05,0.06]	0.6 ± 0.02	0.004 ± 0.01 [−0.05,0.06]	0.62 ± 0.04	−0.01 ± 0.02 [−0.07,0.04]	0.47
Compression	0.62 ± 0.04	0.63 ± 0.04	−0.01 ± 0.01 [−0.05, 0.02]	0.61 ± 0.05	0.01 ± 0.01 [−0.03,0.04]	0.62 ± 0.04	0.004 ± 0.01 [−0.03,0.04]	0.61 ± 0.04	0.005 ± 0.02 [−0.03,0.04]	0.08
Walk	0.6 ± 0.05	0.62 ± 0.04	0.03 ± 0.01 [−0.07,0.01]	0.62 ± 0.06	−0.02 ± 0.01 [−0.07,0.01]	0.6 ± 0.04	−0.01 ± 0.01 [−0.05,0.03]	0.6 ± 0.04	−0.004 ± 0.01 [−0.04,0.04]	0.47
Mean blood flow, mL/min										
Continuous	103.2 ± 58	60.4 ± 17.3	−42.8 ± 39.0 [−68.2, −17.4]	63.2 ± 17.7	−40.0 ± 45.9 [−70.0, −10.0]	64.4 ± 38.5	−38.8 ± 43.9 [−67.5, −10.1]	56.0 ± 11.6	−47.3 ± 56.8 [−84.4, −10.2]	0.75
Compression	89.7 ± 39.1	84.2 ± 35.2	−5.5 ± 42.9 [−33.5, 22.4]	71.6 ± 31.5	−18.1 ± 21.5 [−32.2, −4.1]	69.7 ± 25.7	−20.0 ± 23.4 [−35.3, −4.8]	61.5 ± 35.3	−28.2 ± 26.0 [−46.2, −10.2]	0.14
Walk	63.1 ± 19.1	59.6 ± 24.2	−3.5 ± 17.7 [−15.1, 8.1]	56.6 ± 23.9	−6.5 ± 17.0 [−17.6, 4.6]	71.2 ± 26.3	8.2 ± 23.9 [−7.4, 23.8]*†	50.5 ± 24.6	−12.6 ± 23.6 [−28.0, 2.8]	0.04
Mean blood velocity, cm/s										
Continuous	6.3 ± 3.3	4.2 ± 1.0	−2.1 ± 2.5 [−3.6, −0.6]	4.1 ± 1.4	−2.1 ± 2.6 [−3.8, −0.5]	4.0 ± 2.0	−2.3 ± 2.7 [−4.0, −0.7]	3.6 ± 1.3	−2.6 ± 3.1 [.93,4.4]	0.9
Compression	5.7 ± 1.9	5.3 ± 1.4	−0.5 ± 1.9 [−1.7, 0.7]	5.1 ± 2.2	−0.6 ± 1.7 [−1.7, 0.4]*	4.6 ± 1.9	−1.2 ± 1.9 [−2.4, 0.0]	4.2 ± 1.7	−2.4 ± 2.2 [−2.9, −0.2]*	0.03
Walk	4.2 ± 1.4	4.6 ± 1.9	0.4 ± 0.9 [−0.3, 1.1]	3.9 ± 1.5	−0.3 ± 0.8 [−0.8, 0.2]	4.3 ± 1.4	**0.1 ± 1.3 [−0.8, 0.9]‡**	3.7 ± 1.3	−0.5 ± 1.4 [−1.3, 0.4]	0.08

Values are means ± SD [95% confidence intervals]. Heart rate (HR), mean arterial pressure (MAP), diameter, mean blood flow volume, and mean velocity values with overall change from baseline are shown. Significant values are shown in boldface: *significant difference within group; †significant difference from continuous group (*P* = 0.04); ‡significant difference from continuous group (*P* = 0.01).

#### Blood flow volume.

**Figure 4.**
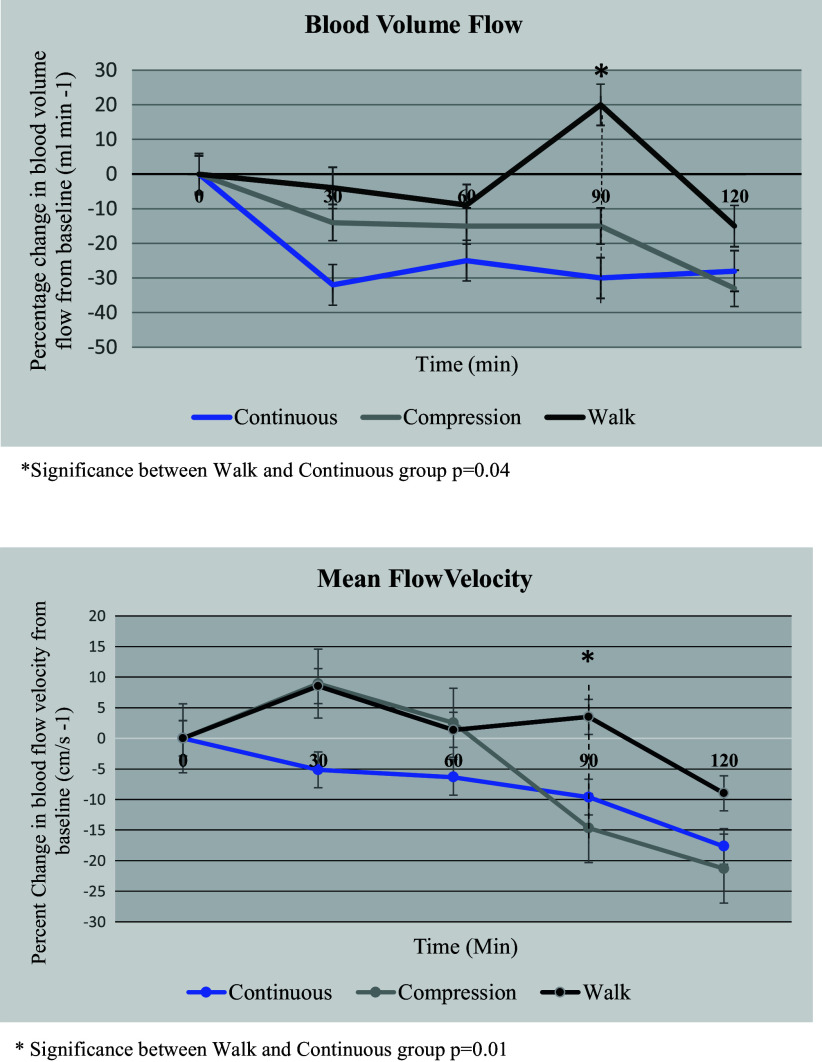
Blood volume (*top*) and mean blood velocity (*bottom*) results. *P = 0.04 (blood volume) and *P* = 0.01 (mean blood velocity), significance between walk and continuous groups.

As shown in Fig. 4, blood flow volume decreased by 38% after 90 min of continuous play while sitting compared with a 22% decrease in blood flow volume in the compression group and a 13% increase in the walking group after 30 min after a 6-min walk.

Blood flow volume decreased by 46% after 120 min of continuous play while sitting compared with a 31% decrease in blood flow volume in the compression group and a 20% decrease in the walking group after 60 min after a 6-min walk.

#### Blood flow velocity.

Blood flow velocity decreased by 37% after 90 min of continuous play while sitting compared with a 19% decrease in blood velocity in the compression group and a 2% increase in the walking group after 30 min after a 6-min walk.

Blood flow velocity decreased by 43% after 120 min of continuous play while sitting compared with a 26% decrease in blood velocity in the compression group and a 12% decrease in the walking group after 60 min after a 6-min walk (Fig. 4).

### Exit Survey

The results revealed that >67% of participants believed that the 6-min walk break had a positive impact on their gaming performance, whereas only 11% felt the compression garments positively impacted performance. Interestingly, >78% of participants agreed that they would consider wearing compression garments in the future, regardless of the way it impacted on gaming. It is important to note that these findings are specific to the participants in this study and may not generalize to the broader gaming population. However, they provide valuable insights into how different interventions are perceived by gamers in terms of their impact on gaming performance (Table 3).

**Table 3.** Player perceptions

Inquiry	%
How did the 6-min walk break impact your game performance?	
Positively	66.7
Negatively	0
No impact	33.3
I enjoyed the 6-min walk.	
Strongly agree	33.3
Agree	44.4
Neither agree/disagree	22.2
How do you feel the compression garments impacted your gaming performance?	
Positively	11.1
Negatively	22.2
No impact	33.3
I enjoyed wearing the compression garments.	
Agree	55.6
Neither agree/disagree	33.3
Disagree	11.1
I would consider wearing compression garments in the future.	
Agree	77.8
Disagree	11.1
Strongly disagree	11.1
The color and look of the compression wear matters to me.	
Neither agree/disagree	33.3
Disagree	55.6
Strongly disagree	11.1
I would wear compression sleeves if it was part of my team uniform.	
Strongly agree	22.2
Agree	77.8

## DISCUSSION

This study presents novel evidence that integrating light-intensity walking breaks during gaming sessions can attenuate the deleterious effects of prolonged sitting on blood flow volume and blood flow velocity more than compression garments. Although this is the first study to investigate the effects of physical activity breaks and compression garments in the gaming population, prior research has probed the hemodynamics of extended sitting in nongaming populations.

In a sitting position the velocity of blood flow in the upward direction (against gravity) is reduced. After 3 h of uninterrupted prolonged sitting, femoral artery blood flow can decline up to 50% (25, 26). Even in children 7–10 yr of age, femoral artery blood flow decreased by 33% (16). This decreased blood flow disturbs homeostasis and can result in blood pooling in the lower extremities, creates procoagulant changes in the blood and endothelial activation, and is the primary cause of DVT. During air travel, Hitos et al. (3) found a 41% decrease in blood flow velocity and 42% in blood volume after 100 min of sitting. Similar changes were discovered in the present study after 120 min of continuous gaming, but they improved when wearing compression sleeves, and they improved even more after a 6-min walk at 60 min (see Fig. 4).

In a recent study by DiFrancisco-Donoghue et al. (27), it was reported that the average collegiate gamer sits 4–8 h daily while practicing or competing in video game play. These extended hours of sitting and play time can vary extensively among recreational gamers. Lippi et al. (6) reported >20 documented cases of computer-related thrombosis events. The ages spanned from as young as 12 to 68 yr, and gaming time spanned from 3–4 h daily to 80 h of consecutive play. In >38% of reported cases the gamers were in their teens (6). It should be noted that it is uncommon to observe a DVT in a person so young and without any recognized risk factors, but in this case the doctors blame continual computer use and atypical positioning.

### Compression

Compression stockings are often recommended as a preventive measure for individuals at risk of DVT, especially during long periods of inactivity or after certain medical procedures. The suggested mechanism of graduated compression is that it leads to a reduction in the cross section of the venous system in the lower limb, enhancing the calf muscle pump’s ability, which may result in an increase in blood velocity and blood flow (2, 28). The pressure and fit of compression gear may vary depending on the individual. Modern compression gear typically uses a technique known as “graduated compression,” where the highest amount of pressure is applied to the most distal parts of the body (such as the ankles for lower body compression) and gradually decreases as it moves up toward the body. Compression wear pressure is measured in millimeters of mercury (mmHg), with light compression ranging from 18 to 21 mmHg, moderate compression ranging from 23 to 32 mmHg, strong compression ranging from 34 to 46 mmHg, and very strong compression of >49 mmHg (29). Most over-the-counter athletic compression garments range from 18 to 21 mmHg in pressure. The recommended compression level for DVT prevention is typically within the range of 15–30 mmHg, with higher levels of compression often suggested for individuals at higher risk (2). In this study the compression used was within this recommended range, ranging from 16.7 to 25.4 mmHg.

Although the study found that wearing compression stockings resulted in moderate improvements at 120 min compared with not wearing them, the results from an active break were superior, suggesting that taking an active break, such as getting up and moving around, may be more effective in reducing the risk of DVT compared with relying solely on compression stockings.

It is important to note that each individual’s risk factors and circumstances may vary, and the effectiveness of compression stockings can depend on various factors. It is always advisable to consult with a health care professional for personalized advice regarding DVT prevention and the use of compression stockings.

Further studies are needed to explore the potential effects of varying degrees of compression on hemodynamics, as well as the potential benefits and risks of compression garments in various contexts.

### Physical Activity

The Centers for Disease Control and Prevention (CDC) recommends walking or standing every 2–3 h during air travel (30). Carter et al. (15) conducted a scientific investigation that revealed how less frequent breaks of longer duration can enhance blood flow to a greater extent than more frequent, shorter breaks. Specifically, the study examined the effects of implementing 2-min walking breaks every 30 min versus an 8-min break occurring at the 120-min mark (15). The results demonstrated that after 4 h of uninterrupted sitting the 8-min walking break led to a significant improvement in blood flow (15). It is important to note that there are distinctions between this study and the present one. The previous study used a motorized treadmill for walking at a higher intensity compared with the present study, which used level ground and a lower intensity. Several studies have demonstrated that a 3-min light walking break at 30 min can increase popliteal blood flow by up to 30% and improve cerebral blood flow (14, 15, 28). During a physically active break, the skeletal muscle undergoes cyclic contractions and relaxations, facilitating the circulation of blood throughout the body and overcoming gravitational forces. The optimal type, intensity, and frequency of physical activity required to counteract the detrimental effects of prolonged sitting can vary based on individual characteristics. The transient benefits of a 6-min walk break, persisting for only 30 min, prompt an exploration into the underlying mechanisms of physical activity’s impact on vascular blood flow and recovery. The intensity and duration of exercise are crucial determinants in these dynamics, with significant implications for cardiovascular and vascular recovery (29, 31). High-intensity exercise markedly increases oxygen demand, leading to a substantial rise in blood flow, predominantly to active muscles. This is facilitated by an increase in cardiac output, driven by elevated heart rate and stroke volume, and is further amplified by vasodilation in active muscle vasculature, mediated by factors such as nitric oxide release (27, 30).

Conversely, low-intensity exercise, as implemented in our study with a very light intensity indicated by an RPE of 9.5 on the Borg scale, results in a more modest increase in blood flow. This lesser demand places reduced strain on the cardiovascular system. The exercise recovery phase involves a gradual normalization of heart rate, blood pressure, and vasodilation, which are also reflective of cardiovascular fitness (27). It is also important to note that the effects of physical activity breaks may vary among gamers, who may experience elevated levels of sympathetic activity, resulting in increases in physiological parameters such as heart rate, respiratory rate, and minute ventilation (29). The findings from this study suggest that a low-intensity 6-min walking break can improve blood hemodynamics for a duration of up to 30 min. However, interventions to reduce sitting time must be practical and applicable in real-life situations (32). Taking short frequent breaks every 30 min during an esports game may not be practical or feasible, especially considering the nature of certain games. Therefore, for this study, we opted to introduce a break at the 60-min mark, with a cumulative duration of 6 min. This approach allows for some interruption in game play while acknowledging the unique demands and dynamics of esports, striking a balance between the need for breaks and the uninterrupted flow required for effective gaming experiences. The objective of this study was to create a realistic environment for gamers and to design an intervention that can be easily implemented in such a setting. To achieve this, the walking intervention’s intensity was set at a perceived exertion level of 6–9 on a Borg scale, on a flat indoor surface that corresponds to light activity such as walking to the bathroom and can be performed in any environment.

Previous research on the efficacy of incorporating walking breaks into daily routines has primarily centered around younger individuals and office workers. However, it is important to consider that the effects of physical activity breaks may vary among gamers, who may experience elevated levels of sympathetic activity, resulting in increases in physiological parameters such as heart rate, respiratory rate, and minute ventilation (12).

### Exit Survey

One of the major challenges encountered by competitive gamers at both the collegiate and professional levels is the need to educate players about health and performance. Coaches often face resistance when introducing new protocols and motivating players to adopt strategies that enhance their well-being and performance. However, the results of our exit survey are highly encouraging, as the majority of participants expressed a preference for the walking break and believed that it positively affected their performance. Approximately 78% perceived the walking break as a positive experience, and >66% felt that it had a beneficial impact on their gaming performance. Almost 90% of gamers reported enjoying wearing the compression garments, and >88% expressed a willingness to consider wearing them in the future. These findings indicate promising interventions that can be easily incorporated into the daily lives of gamers and gaming teams.

### Limitations

Although this study used self-reported recall on physical activity and game play, it should be noted that there may be recall bias due to social desirability. Further limitations of our study were the absence of recorded data regarding activity the day before testing, the use of oral contraceptives, and the reliance on self-reported measures for daily fluid, sodium, and food intake. We controlled hydration during the gaming sessions (no fluid intake was allowed during any session). Fluid intake was not restricted before testing except to ask subjects to try and drink a similar amount on each testing day.

### Clinical Implications

Research concerning esports players is currently in its infant stage. This study represents an initial attempt to implement active breaks and compression wear with a specific frequency and dosage, aiming to aid coaches and clinicians in developing guidelines that mitigate the risk of DVTs and counteract the detrimental impacts of extended periods of sitting during gaming sessions while minimizing gaming disruptions, based on prior research that highlighted improved executive function in gamers following a 6-min walking break at the 60-min interval and our present observations of increased blood volume and velocity in lower limb vasculature after a 6-min period of physical activity. These findings collectively support the recommendation of incorporating brief, 6-min active breaks during gaming sessions, at intervals of 60 min. Additionally, for enhanced performance and health benefits, it is advisable, when feasible, to schedule these active pauses every 30 min (20).

In conclusion, compression stockings demonstrated potential benefits for blood hemodynamics, underscoring the significance of integrating brief physical activity breaks into gaming routines. Short breaks, even just a few minutes, positively impact blood flow and cardiovascular health for those spending extended periods gaming. Video gamers favor walking breaks, believing they enhance performance. By adopting these simple measures, gamers can mitigate health risks linked to prolonged sitting and enhance overall well-being. Given these results, further research into higher intensities and varying durations of physical activity, particularly those aligning with the demands of esports competitions, is warranted. This would potentially extend the benefits of physical breaks in such settings and contribute to a more comprehensive understanding of exercise-induced hemodynamic responses.

## DATA AVAILABILITY

Data will be made available upon reasonable request.

## GRANTS

This study was supported by Fnatic, Ltd.

## DISCLOSURES

The authors disclose a professional partnership with Fnatic, Ltd., who helped support this project. J.D.-D. was supported by Fnatic, Ltd., who helped support this project by providing keyboards and gaming mouse pads to participants. None of the other authors has any conflicts of interest, financial or otherwise, to disclose.

## AUTHOR CONTRIBUTIONS

J.D.-D. and P.C.D. conceived and designed research; J.D.-D., K.B., T.L., O.B., and H.Z. performed experiments; J.D.-D., K.B., T.L., and P.C.D. analyzed data; J.D.-D., K.B., T.L., H.Z., and P.C.D. interpreted results of experiments; J.D.-D. prepared figures; J.D.-D., K.B., and P.C.D. drafted manuscript; J.D.-D., T.L., O.B., and P.C.D. edited and revised manuscript; J.D.-D., K.B., H.Z., and P.C.D. approved final version of manuscript.
